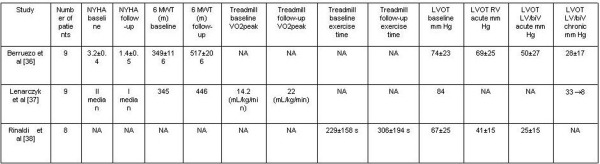# Biventricular / Left Ventricular Pacing in Hypertrophic Obstructive Cardiomyopathy: An Overview

**DOI:** 10.1016/s0972-6292(16)30503-4

**Published:** 2012-05-20

**Authors:** Radu Vatasescu, Reinder Evertz, Lluis Mont, Marta Sitges, Josep Brugada, Antonio Berruezo

**Affiliations:** 1 Cardiology Department, Clinic Emergency Hospital, Bucharest, Romania; 2 Arrhythmia Section, Cardiology Department, Thorax Institute, Hospital Clinic, Barcelona, Spain

**Keywords:** hypertrophic obstructive cardiomyopathy, intraventricular gradient, biventricular pacing, reverse remodelling

## Abstract

Hypertrophic cardiomyopathy (HCM) is an autosomal dominant inherited genetic disease characterized by compensatory pathological left ventricle (LV) hypertrophy due to sarcomere dysfunction. In an important proportion of patients with HCM, the site and extent of cardiac hypertrophy results in severe obstruction to LV outflow tract (LVOT), contributing to disabling symptoms and increasing the risk of sudden cardiac death (SCD). In patients with progressive and/or refractory symptoms despite optimal pharmacological treatment, invasive therapies that diminish or abolish LVOT obstruction relieve heart failure-related symptoms, improve quality of life and could be associated with long-term survival similar to that observed in the general population. The gold standard in this respect is surgical septal myectomy, which might be supplementary associated with a reduction in SCD. Percutaneous techniques, particularly alcohol septal ablation (ASA) and more recently radiofrequency (RF) septal ablation, can achieve LVOT gradient reduction and symptomatic benefit in a large proportion of HOCM patients at the cost of a supposedly limited septal myocardial necrosis and a 10-20% risk of chronic atrioventricular block. After an initial period of enthusiasm, standard DDD pacing failed to show in randomized trials significant LVOT gradient reductions and objective improvement in exercise capacity. However, case reports and recent small pilot studies suggested that atrial synchronous LV or biventricular (biV) pacing significantly reduce LVOT obstruction and improve symptoms (acutely as well as long-term) in a large proportion of severely symptomatic HOCM patients not suitable to other gradient reduction therapies. Moreover, biV/LV pacing in HOCM seems to be associated with significant LV reverse remodelling.

## Introduction

Hypertrophic cardiomyopathy (HCM) is an autosomal dominant inherited genetic disease characterized by compensatory LV hypertrophy mainly due to sarcomere dysfunction. Prevalence of the disorder in the general population is estimated to be 0.2% [1]. A subset of patients with HCM has hypertrophic obstructive cardiomyopathy (HOCM), in which systolic septal bulging into the LVOT, malposition of the anterior papillary muscle, with enlarged posterior mitral leaflet and hyperdynamic LV contraction and drag forces, through a Venturi effect, provoke systolic anterior motion of the anterior leaflet of the mitral valve (SAM), contributing to the creation of the LVOT gradient. The degree of LVOT obstruction is generally variable, with only a minority of patients presenting significant LVOT gradient at rest. In the majority of HOCM the LVOT obstruction is only "latent" or "provocable" by stimuli such as exercise, drugs (amyle nitrate), Valsalva maneuver and postextra- systolic potentiation [2]. Resting (basal) obstruction in HOCM is an independent predictor of adverse clinical consequences such as progressive heart failure and cardiovascular death, including sudden cardiac death (SCD) [3,4].

## Overview of the treatment in HOCM

Medical therapy is at least partially effective in the majority of HOCM patients [5] and consists of beta-blockers, calcium channel blockers and disopyramide, the later being of particular benefit in patients with associated atrial fibrillation due to its atrial antiarrhythmic properties.

In severely symptomatic HOCM patients despite optimal medical treatment and with significant resting or provocable LVOT obstruction (with gradient ≥30 mm Hg at rest or ≥50 mm Hg during exercise) non-pharmacologic treatment is indicated [5]. Invasive therapies should be able to alleviate LVOT obstruction either by increasing LVOT systolic diameter and/or by reducing/eliminating SAM. Surgical septal myectomy is the gold standard in this respect, being able to concomitantly eliminate anomalies of mitral valve apparatus by concomitant mitral valve repair.

Surgical myectomy is effective in >90% of patients, with long-term survival similar to that of the general population [6] and a potential association with a reduction in SCD [7]. In experienced centres perioperative mortality is <1% and risk of atrioventricular bock (AVB) is <3% [8]. The rate of perioperative mortality and major complications may be significantly higher in patients with significant co-morbidities, particularly in those over 65 years old [9,10].

Percutaneous alcohol septal ablation (ASA) can achieve significant LVOT gradient reduction and symptomatic benefit in approximately 80% of HOCM patients, with long-term survival comparable with surgical myectomy. ASA-related mortality is 1.5-3% and there is a 10-20% risk of chronic AVB [11-13]. Moreover, 9 to 13% of patients require a second procedure and 3-5% surgical myectomy [11,12]. There is also ongoing concern about SCD/ventricular arrhythmia due to ASA-induced septal myocardial necrosis, which might in the long term behave as an arrhythmogenic substrate. Although to date most of the reports suggest that ASA is not associated with an increase in SCD/ventricular arrhythmia [14], it is worrisome that successful LVOT gradient reduction by ASA does not reduce the risk for malignant arrhythmias in high-risk HOCM patients [15,16].

Percutaneous radiofrequency (RF) septal ablation is a recently introduced method that can relieve LVOT gradient through RF-induced necrosis in the basal septum. It has the theoretical advantages that it is not dependent on coronary artery anatomy and can precisely identify His bundle and its branches and therefore avoid their damage. However, recently published small series showed persistent significant LVOT gradient in more than 20% of the HOCM patients, with significant perioperative mortality and life-threatening complications (including malignant ventricular arrhythmia), and a > 20% rate of AVB [17,18]. Moreover, for the moment data on long-term risk for SCD/ventricular arrhythmia are lacking.

### DDD pacing in HOCM

Surgical myectomy and ASA can carry unacceptable risks and/or are not suitable in selected subsets of patients with HOCM and severe LV obstruction. Dual-chamber (DDD) pacing with a short atrioventricular delay, a widely available alternative, initially raised interest for the treatment of LVOT obstruction. Two main mechanisms, systolic and diastolic, are known to be involved in reducing LV gradient with DDD-right ventricular apex (RVA) pacing. The systolic mechanism relies on the paradoxical motion of the interventricular septum induced by altered LV depolarization, which delays contraction of the basal interventricular septum and therefore increases the systolic dimension of the LVOT [19] with secondary reduction in systolic anterior motion of the mitral valve. The diastolic mechanism relies on the improved LV filling due to optimized timing of atrial systole [20]. Observational studies and a non-blinded randomized trial have suggested that DDD pacing produces gradient reduction, with symptomatic and functional benefit [21-23]. Subsequent single and multicentre randomized trials demonstrated average LVOT gradient reductions of only 50 percent and no improvement in exercise capacity [24-26], suggesting a placebo effect for the symptomatic improvement [24,27]. One possible explanation for this limited response could be that the change in motion of the interventricular septum induced by RVA pacing is too small to significantly reduce the LVOT gradient, because many HOCM patients have a very rapid AV conduction [28] and/or the RVA sites are at a distance away from the LV apex [29]. Moreover, recent long-term follow-up data suggests that DDD pacing in HOCM patients might have a deleterious effect on survival and heart failure by comparison with conservative management [30]. Based on some of these data current guidelines assign a class IIb indication for DDD pacing in HOCM patients without a bradycardia indication [5,31].

## The case of atrial synchronous LV or biventricular pacing in HOCM

A small number of case reports showed that atrial synchronous LV or biventricular pacing (biV) might further reduce the LV pressure gradient and improve symptoms in HOCM, initially in patients with intraventricular conduction delay [32]. Later on, LV/biV pacing showed a significant LVOT gradient reduction superior to RVA pacing in HOCM patients without intraventricular conduction delay [29,33-35]. Recently, published data from three small studies suggest that LV/biV pacing might be efficient for LVOT gradient reduction and symptom improvement in a large proportion of HOCM patients not suitable for myectomy or ASA (Table 1) [36,39].

### Mechanisms of LVOT gradient reduction with LV/biV pacing

In contrast to RVA pacing, LV/biV pacing might be able to induce a supplementary reduction in LVOT gradient by an alteration in the contraction of a larger area of the LV. Alternatively, the reversed LV depolarization sequence [35] caused by pre-excitation of the LV posterolateral/lateral wall during LV/biV pacing may activate the longitudinally oriented epicardial fibres earlier, thereby advancing lateral wall longitudinal displacement with regard to interventricular septal longitudinal displacement [36] and potentially even slightly stretching the latter. This will change the pre-ejection shape and diameter of the LVOT and mitral valve, which, together with the induced reduction in septal systolic displacement, can reduce the intraventricular gradient. Support for the latter hypothesis comes from a case report showing that DDD RVA epicardial pacing for complete AV block after septal myectomy induced SAM and LVOT gradient, while LV lateral wall pacing completely abolished it [39]. SAM disappearance was also observed in the majority of HOCM patients after 6 months of LV/biV pacing [37].

### LV/biV implant procedure outcome

**Figure 1:**
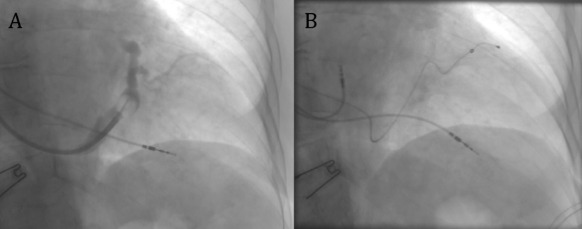
A: RAO projection showing the occlusive venography in a hypertrophic obstructive cardiomyopathy patient; note the extreme tortuousity as well as the reduced diameter of the target coronary sinus ventricular branch. B: The same RAO projection showing the final lead position of the leads (a 4F bipolar lead was used).

Recently published small studies with LV/biV pacing in HOCM showed an acute success rate of transvenous LV lead implantation ranging from 75-80% [36,37] to 100% [39] compared to the 94.4% CRT implantation rate in patients with LV systolic dysfunction [40] or 100% in patients with dilated (end-stage) phase of hypertrophic cardiomyopathy [41]. Causes of LV lead implantation failure were: impossibility to cannulate coronary sinus (CS) ostium [36,37], intense phrenic nerve stimulation despite LV repositioning [37] and CS dissection [37]. Possible explanations are the smaller and/or tortuous coronary sinus and its ventricular branches in concentrically hypertrophied non-dilated LV of HOCM patients (Figure 1), as well as the limited experience of the operators in this population and/or lack of specifically adapted LV leads. It is possible that newer generation LV leads, with smaller diameter (around 4F for bipolar leads) and better trackability could help to increase success rates. A low rate of LV lead or device-related complications have been described on long-term follow-up, although in one of the three small pilot studies the LV lead was extracted in three of eight cases (one due to device infection) [37]. Additionally, in a very recent retrospective analysis of a single-centre ICD registry in patients with HCM, 22% of the CRT-D subgroup showed LV lead-related complications [42].

### Symptoms and functional capacity

Small long-term pilot studies have shown improvements of at least one NYHA functional class with LV/biV pacing [36] even in less symptomatic HOCM patients [37], with a marked and progressive increase in quality of life [36,38] and functional capacity evaluated by 6-minute walk test (6MWT) [36,37], treadmill exercise test [38] or peak oxygen consumption (VO2 peak) [37] (Table 1). In one of the studies a non-uniform response to LV/biV pacing was observed: two out of eight patients were symptomatic non-responders, three responded to biV pacing and three to RVA pacing [38]. However, in this study the optimal pacing configuration was established by the maximum symptomatic benefit observed after a serial crossover comparison between no active pacing vs. RVA pacing or biV pacing. Additionally, in non-responders mean LVOT gradient at rest was less significant (around 25 mm Hg) and in three out of eight patients previous septal reduction therapies failed (ASA in two patients and myectomy in another one) [38]. Other studies decided the optimal pacing configuration to be the one that acutely induces the maximum LVOT gradient reduction [36,37], possibly explaining their higher rate of responders. Overall LV/biV seems to favourably compare with studies on DDD RVA pacing in HOCM, which failed to show significant improvement in functional capacity [24-26].

### LVOT obstruction

**Figure 2:**
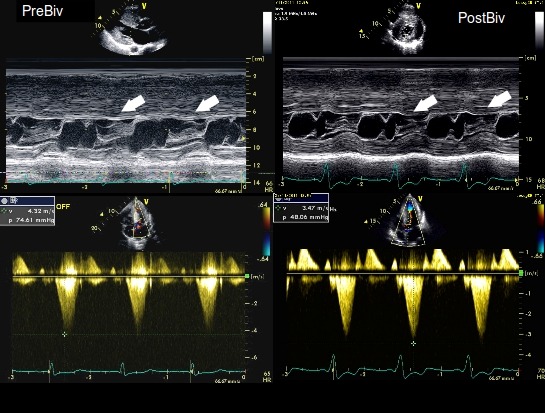
Upper panels show Mmode scans across the left ventricle and the mitral valve before (preBiv) and after atrial synchronous biventricular pacing (postBiv): arrows indicate systolic anterior motion (SAM) of the mitral valve, which touches the septum preBiv and does not contact it postBiv. These changes in the degree of SAM are concomitantly seen with a significant decrease of LVOT gradient (from 76 to 48 mmHg). In this patient, atrial synchronous right ventricular apical pacing did not change the left ventricular outflow tract gradient.

In the overwhelming majority of HOCM patients LV/biV pacing is acutely more effective for gradient reduction than standard RVA pacing: eight out of nine in one study [36] and all nine patients in another [37]. Even when optimal pacing configuration was selected by symptomatic improvement, clinical non-responders and patients selected for RVA pacing still achieved a greater reduction in LVOT gradient with biV pacing. [38] (Table 1). Moreover, LVOT gradient progressively decreased during follow-up, with a mean LVOT gradient at rest below 30 mm Hg at medium term [36,37], reaching almost non-significant levels at three years [37]. Acutely, SAM is still present (although with a significantly reduced contact time; unpublished observations, Dr. Vidal) [36]. Long-term, LV/biV pacing is associated with a continuous reduction of SAM [36] or even complete disappearance in the majority of HOCM patients [37] (Figure 2). This is concordant with a continuous and significant reduction in the degree of mitral regurgitation [36]. Interestingly, one of the studies showed that the optimal atrioventricular and interventricular intervals for maximum LVOT gradient reduction at six months follow-up were different than those at baseline for more than 50% of the patients [37].

### LV reverse remodelling

In contrast to studies with DDD RVA pacing, LV/biV pacing showed that the reduction in LVOT gradient induced a progressive and significant reduction in LV mass (reverse remodelling) [36]. The thinning seems to be significant only for the interventricular septum [36,37], although there was also a trend in the LV posterior wall [36]. This is not surprising, considering that LV hypertrophy in young patients with HOCM is at least partially also secondary to LVOT obstruction [43] and that septal reduction therapies induce LV reverse remodelling, with wall thinning in areas distant from the interventricular septum [44,45].

### LV/biV pacing in HOCM and SCD / ventricular arrhythmia

During long term LV/biV pacing in HOCM there was no SCD/syncope in patients with CRT-P [36,38]. In the group of CRT-D patients, only one experienced appropriate ICD therapies [37]. Although the pooled number of studies with LV/biV pacing in HOCM is small, it is tempting to speculate that this therapy, which is able to reduce LVOT gradient and induce LV reverse remodelling without creating a septal scar, does not increase the SCD/VA risk. Data from CRT studies in patients with LV systolic dysfunction also demonstrate that the risk of SCD/VA is reduced in the presence of significant LV reverse remodelling [46,47].

### Limitations

The small number of patients included in studies with LV/biV pacing in HOCM as well as the fact that all of them are observational and uncontrolled (i.e., a placebo effect of pacing cannot be excluded) requires caution in interpreting the results. However, the differences observed in comparison with standard DDD RVA pacing warrants further research. Considering the relatively large number of HOCM patients submitted to cardioverter-defibrillator implantation for primary or secondary prevention, data for such analysis should be readily available.

### Conclusion

In selected patients with HOCM, LV/biV pacing is feasible and usually the best configuration for gradient reduction. Overall, LV/biV pacing in patients with HOCM significantly and progressively improves functional capacity and quality of life. It may also induce LV reverse remodelling.

## Figures and Tables

**Table 1:**